# Stability, survival, and tolerability of a 4.5-mm-wide bone-anchored hearing implant: 6-month data from a randomized controlled clinical trial

**DOI:** 10.1007/s00405-015-3593-x

**Published:** 2015-03-20

**Authors:** Rik C. Nelissen, Christine A. den Besten, Emmanuel A. M. Mylanus, Myrthe K. S. Hol

**Affiliations:** Department of Otorhinolaryngology, Donders Institute for Brain, Cognition and Behaviour, Radboud University Medical Center, P.O. Box 9101, 6500 HB Nijmegen, The Netherlands

**Keywords:** Bone-anchored hearing aid, Bone-anchored hearing system, Baha, Ponto, Bone conduction, Implant stability quotient (ISQ), Resonance frequency analysis (RFA), Early loading, Implant survival, Quality of life

## Abstract

The objective of this study was to compare the stability, survival, and tolerability of 2 percutaneous osseointegrated titanium implants for bone conduction hearing: a 4.5-mm diameter implant (test) and a 3.75-mm diameter implant (control). Fifty-seven adult patients were included in this randomized controlled clinical trial. Sixty implants were allocated in a 2:1 (test–control) ratio. Follow-up visits were scheduled at 7, 14, 21, and 28 days; 6 and 12 weeks; and 6 months. At every visit, implant stability quotient (ISQ) values were recorded by means of resonance frequency analysis (RFA) and skin reactions were evaluated according to the Holgers classification. Implants were loaded with the bone conduction device at 3 weeks. Hearing-related quality of life was evaluated using the Abbreviated Profile of Hearing Aid Benefit (APHAB), the Glasgow Benefit Inventory (GBI), and the Glasgow Health Status Inventory (GHSI). ISQ values were statistically significantly higher for the test implant compared to the control implant. No implants were lost and soft tissue reactions were comparable for both implants. Positive results were reported in the hearing-related quality of life questionnaires. These 6-month results indicate that both implants and their corresponding hearing devices are safe options for hearing rehabilitation in patients with the appropriate indications. Loading at 3 weeks did not affect the stability of either implant.

## Introduction

Percutaneous osseointegrated titanium implants have been used to attach vibrating bone conduction devices to the temporal bone since 1977 [1]. Both implants and devices, as well as the indications for application, have been studied extensively [2, 3]. The clinical outcomes of these implants have been reported in large populations: long-term implant survival rates vary between 81.5 and 98.4 %, while complications generally involve soft tissue inflammation [4–6]. Severe complications are rare [4, 5].

Recently, the designs of these bone-anchored hearing implants have evolved to include wider diameters, based on the known advantages of wider implants in dentistry [7]. These 4.5-mm-wide implants provide a larger contact surface between the implant and the bone compared to the 3.75-mm-wide implants of the previous generation, which results in higher reported implant stability quotients (ISQ) and high implant survival rates [8, 9]. Moreover, wider implants appear to have higher levels of initial stability, which allows for early loading of the implant with the device. Loading wider implants has been reported to be safe at 3 weeks after surgery [10].

The current randomized controlled clinical trial investigated ISQ, implant survival, and soft tissue tolerability of a new wide implant in comparison to a previous generation implant in the first 6 months after implantation. Early loading of both implants was studied, with all implants loaded at 3 weeks. Subjective benefits of the bone conduction system were investigated using quality of life questionnaires.

## Methods

### Implants and patients

**Fig. 1:**
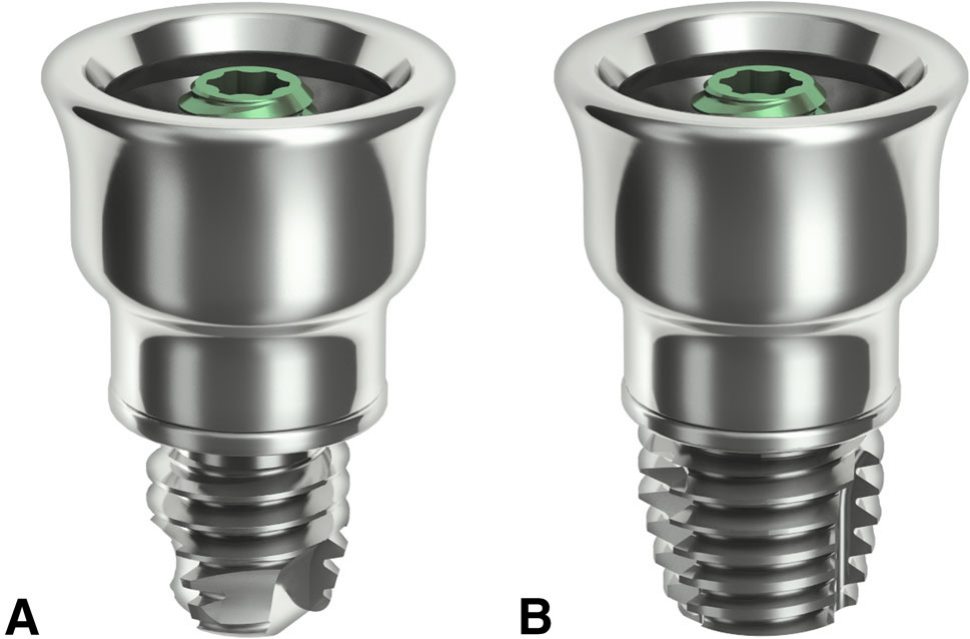
Control (**a**) and test (**b**) implants with abutments

The test implant was the wide Ponto implant (diameter 4.5 mm, length 4 mm) and the control implant was the previous generation Ponto implant (diameter 3.75 mm, length 4 mm). Both implants used the same 6-mm abutment. The implants and abutments are developed and manufactured by Oticon Medical AB (Askim, Sweden) and are displayed in Fig. 1.

Out of all of the patients indicated for a percutaneous bone conduction device in our center, 57 adult patients with a total of 60 implants were consecutively included. Eligibility criteria were as follows: indication for a percutaneous implant; age of 18 years or older; bone thickness of at least 4 mm at the implant site; written informed consent given; abutment of 6 mm required (not longer); ability to participate in follow-up visits; no history of psychiatric diseases; no mental disabilities; no presumed doubt, for any reason, that the patient would not be able to attend all follow-up visits; no presence of diseases or use of treatments known to compromise bone quality at the implant site (e.g., radiotherapy, osteoporosis, diabetes mellitus).

### Study design

The current study was designed as an open randomized controlled clinical trial in our tertiary referral center. The primary outcome parameter was implant stability measured as ISQ low values in the first 6 months after implantation. Secondary objectives were to compare ISQ high values in the same period, ISQ low and high values at all visits, time to stability dip (in ISQ low) if applicable, implant survival, soft tissue reactions during all visits, and quality of life outcomes.

The sample size was based on the primary efficacy variable. A weighted average of ISQ low values during the 6-month follow-up period was obtained by the mean area under the curve (AUC) calculation using the trapezoid rule with all ISQ low measurements over the first 6 months. Data from a similarly designed previous study [11] were used for the sample size calculation. An expected difference of 4.5 in the mean AUC of the ISQ low values of the test and the control groups, with unequal standard deviations of 2.8 and 5.5, respectively, were used for determining the sample size. A 2-sided *t* test with Satterthwaite’s correction for unequal variances was performed. For a power of 90 %, significance level of 0.05, and randomization ratio of 2:1, a total of 60 implants needed to be included.

Randomization was performed in a 2:1 ratio (test–control). A computer-generated list of random allocations was used. The group assignments were enclosed in sequentially numbered opaque sealed envelopes. The randomization was blinded to the patients and investigators until the surgery was performed. Patients were allocated in consecutive order. Blinding of the investigators after the group assignments were made was not feasible because the appearances of the implants and instruments used during surgery were clearly different. Because most patients were operated under local anesthesia, the patients were also not blinded.

Implants and abutments were placed in a single-stage surgical procedure. The linear incision technique with subcutaneous tissue reduction was applied in all cases [12]. Implants were alternately placed within or posterior to the incision line. In accordance with the study protocol, follow-up visits were scheduled at 7, 14, 21, and 28 days; 6 and 12 weeks; and 6 months. At each visit, resonance frequency analysis (RFA) was used to establish the implant stability quotient (ISQ). RFA uses magnetic pulses generated by the Osstell ISQ device (Osstell AB, Göteborg, Sweden) to excite the SmartPeg (type 55) that is connected to the abutment, which leads to vibration of the implant–abutment system. The intensity of these vibrations is analyzed by the device that computes the ISQ, which is an indication of the rigidity of the implant–bone interface [13]. Perpendicular measurements result in an ISQ high value and an ISQ low value. At each visit, the skin status was also assessed according to the Holgers classification [14]. Three weeks after surgery, the patients were fitted with the bone conduction device. The benefit of the bone conduction system was assessed using 3 questionnaires: the Abbreviated Profile of Hearing Aid Benefit (APHAB) [15], the Glasgow Benefit Inventory (GBI), and the Glasgow Health Status Inventory (GHSI) [16]. APHAB and GHSI outcomes were only included in the analysis when both the baseline screening before implantation and the 6-month evaluation had been completed. In cases where patients used hearing aids at the baseline evaluation, they were asked to complete the baseline questionnaire both for the aided and unaided conditions. The unaided condition was used as the baseline measurement for analyzing the benefit of the bone conduction system at 6 months.

### Statistical analysis

Data management and statistical analyses were performed by external data managers and biostatisticians (Statistiska Konsultgruppen, Göteborg, Sweden) according to a predefined statistical analysis plan.

For comparisons between the test and control groups, Mann–Whitney *U* tests were used for all continuous variables, Mantel–Haenszel Chi-square tests were used for all ordered categorical variables, Fisher’s exact test was used for all dichotomous variables, and Chi-square tests were used for all non-ordered categorical variables. For changes over time, Wilcoxon signed rank tests (continuous variables) and sign tests (order categorical variables, dichotomous variables) were used. Groups were compared according to the intention-to-treat principle. For subjects lost to follow-up, last observation carried forward was used for ISQ measurements in the AUC calculations.

For implant variables, bilaterally implanted patients who received both a control and a test implant were included in both analyses. Patients who received 2 test or 2 control implants were represented by the mean of the 2 measurements for continuous variables or the worst value for categorical variables. For patient variables, bilaterally implanted patients who received both control and test implants were included in descriptive statistics but excluded in formal analyses on the patient level.

All tests were 2 tailed with significance levels of 0.05 and were executed using SAS v9.2 and v9.3 software (SAS Institute Inc., Cary, NC, USA).

### Ethical considerations

This clinical investigation was performed in accordance with the current version of the Declaration of Helsinki (Washington 2002, ISO 14155), Good Clinical Practice (International Conference on Harmonization Good Clinical Practice) and was approved by the local ethical committee.

## Results

### Patients

Fifty-seven patients with a total of 60 bone-anchored hearing implants were included in the randomization, with 39 implants in the test group and 21 in the control group. Surgeries were performed between June 2012 and January 2014. Baseline demographic information is shown in Table 1. No significant differences were found between the test and control populations. Three patients received bilateral implants; 2 of these patients were randomized for both a test and a control implant, and 1 patient received 2 test implants. All randomized patients received their allocated treatment and could be included in the final 6-month analysis.

**Table 1:** Patient demographics and baseline characteristics

Variables	Test (*n* = 38)	Control (*n* = 21)	*p* value
Gender, *n* (%)			
Male	15 (39.5)	9 (42.9)	1.0000
Female	23 (60.5)	12 (57.1)	
Age in years, mean (SD)	53.7 (12.2)	53.0 (16.0)	0.5469
Smoking at baseline, *n* (%)	6 (15.8)	6 (28.6)	0.3511
Body mass index, mean (SD)	25.9 (4.2)	25.4 (4.0)	0.7635
Skin disease, *n* (%)	4 (10.5)	3 (14.3)	0.9176
Indication for bone-anchored hearing implant, *n* (%)			
Acquired conductive/mixed hearing loss	25 (65.8)	17 (81.0)	0.5279
Congenital conductive hearing loss	1 (2.6)	1 (4.8)	1.0000
Single-sided deafness	13 (34.2)	3 (14.3)	0.2018

### Implant stability quotient

**Fig. 2:**
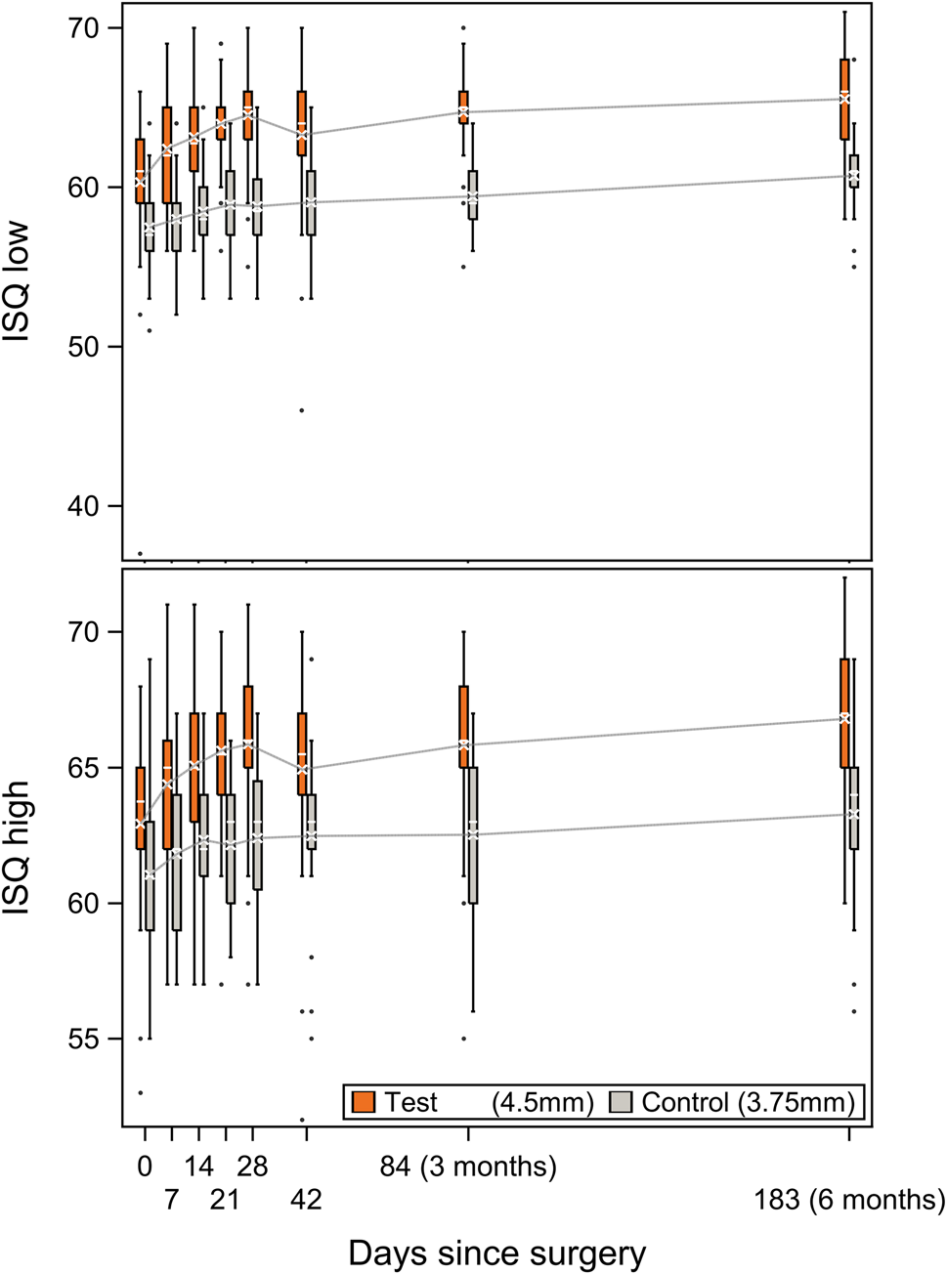
Box-and-whisker plots of ISQ low and ISQ high measurements. The mean (*cross*) and median (*horizontal line*) are defined within each box plot. *Dots* represent outlier values

The mean AUC for ISQ low was 64.4 (SD 3.0; range 55.5–70.1) for test implants (*n* = 38) and 59.5 (SD 2.2; range 55.5–63.5) for control implants (*n* = 21). The difference between these groups of 4.9 ISQ points (95 % CI 3.4–6.4; *p* < 0.0001) was statistically significant. For ISQ high, a difference of 3.2 (95 % CI 1.7–4.7; *p* < 0.0001) was observed during the 6-month follow-up, with a mean AUC of 65.8 (SD 2.7; range 57.0–70.5) for the test implant and 62.6 (SD 2.8; range 56.9–66.8) for the control implant. At all follow-up visits, statistically significant differences in mean ISQs between both groups were recorded. The results are displayed in Fig. 2. The mean increase in ISQ from baseline is statistically significant in both groups; however, the increase in ISQ from baseline for the test implant is statistically significantly stronger compared to the increase for the control implant. The ISQ dip at 42 days for the test implant can be ascribed to a single implant that displayed a very low ISQ (ISQ low 46, ISQ high 52) but remained clinically stable and presented with an ISQ within the normal range at the next follow-up appointment.

No dip in mean ISQ was observed, as the ISQ high and ISQ low values were higher than the baseline ISQ values (at surgery) at all follow-up visits.

Implants were loaded 3 weeks after surgery (with a 2-day range) in all but 1 patient (loaded at 24 days). This early loading moment did not seem to influence ISQ values, as these progressed positively in both implants.

At 6 months, a mean increase in the ISQ low from the time of surgery of 4.5 (SD 4.6; range −4 to 29) was observed for the total group (*n* = 59), which was significantly different from the ISQ low at the time of surgery (*p* < 0.0001). The mean increase was 5.2 (SD 5.0; range −4 to 29) in the test group and 3.2 (SD 3.7; range −3 to 13) in the control group. The mean difference in the increase in ISQ low between both groups was statistically significant (95 % CI −0.5 to 4.5; *p* = 0.03).

### Survival and tolerability

**Fig. 3:**
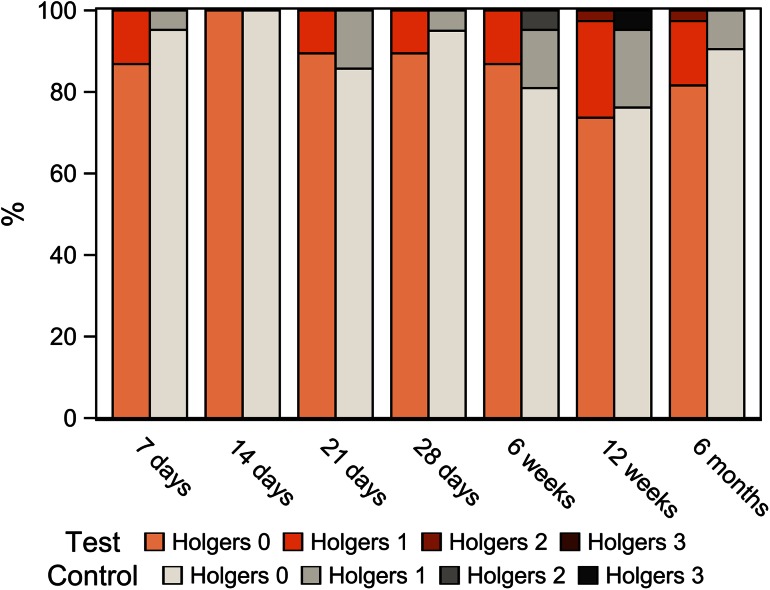
Soft tissue tolerability for test and control groups as a percentage of all visits according to the Holgers classification. Note that only Holgers grade 0–3 are depicted, as no Holgers grade 4 was observed

No implants were lost during the follow-up period. In each experimental group, 1 implant required surgical revision of the soft tissue; 1 patient who suffered from psoriasis presented with insufficient skin healing after surgery and the other patient presented with skin partially overgrowing the abutment. Three implants (7.9 %) in the test group and 2 implants (9.5 %) in the control group developed adverse skin reactions (Holgers grade 2–4). Results related to soft tissue reactions are displayed in Fig. 3. The analysis of soft tissue statuses throughout the follow-up period revealed findings of Holgers grade 0 in 86.8 % (test) and 89.0 % (control) of visits, Holgers grade 1 in 12.1 % (test) and 9.1 % (control) of visits, Holgers grade 2 in 1.1 % (test) and 1.3 % (control) of visits, Holgers grade 3 in 0.0 % (test) and 0.6 % (control) of visits, and no Holgers grade 4 cases over all of the visits. Two out of the 5 patients who presented with adverse skin reactions suffered from skin diseases. Furthermore, no statistically significant differences were noted in other postoperative complications: bleeding or hematoma [1 test (2.6 %) vs 1 control (4.8 %) implant], pain or numbness [4 test (10.5 %) vs 2 control (9.5 %) implants], and wound dehiscence [2 test 5.3 % vs 3 control (14.3 %) implants]. Additionally, skin height did not differ between the groups.

### Quality of life

The GBI questionnaire was completed 12 weeks after surgery. Eight patients completed the questionnaire outside of the defined visit window (mean of 22 days after the planned visit date). These results were still included in the final analysis. No differences were observed in the outcomes between the test and control groups. The results are shown in Table 2.

**Table 2:** Subjective benefit as measured by the GBI

Variables (SD)	Test (*n* = 38)	Control (*n* = 21)	*p* value
Total score	34.2 (19.2)	34.5 (16.6)	0.8384
General subscale	47.8 (25.1)	48.8 (22.5)	0.9223
Social subscale	11.3 (20.8)	9.52 (17.9)	0.9472
Physical subscale	3.1 (15.4)	3.2 (21.5)	0.1571

**Fig. 4:**
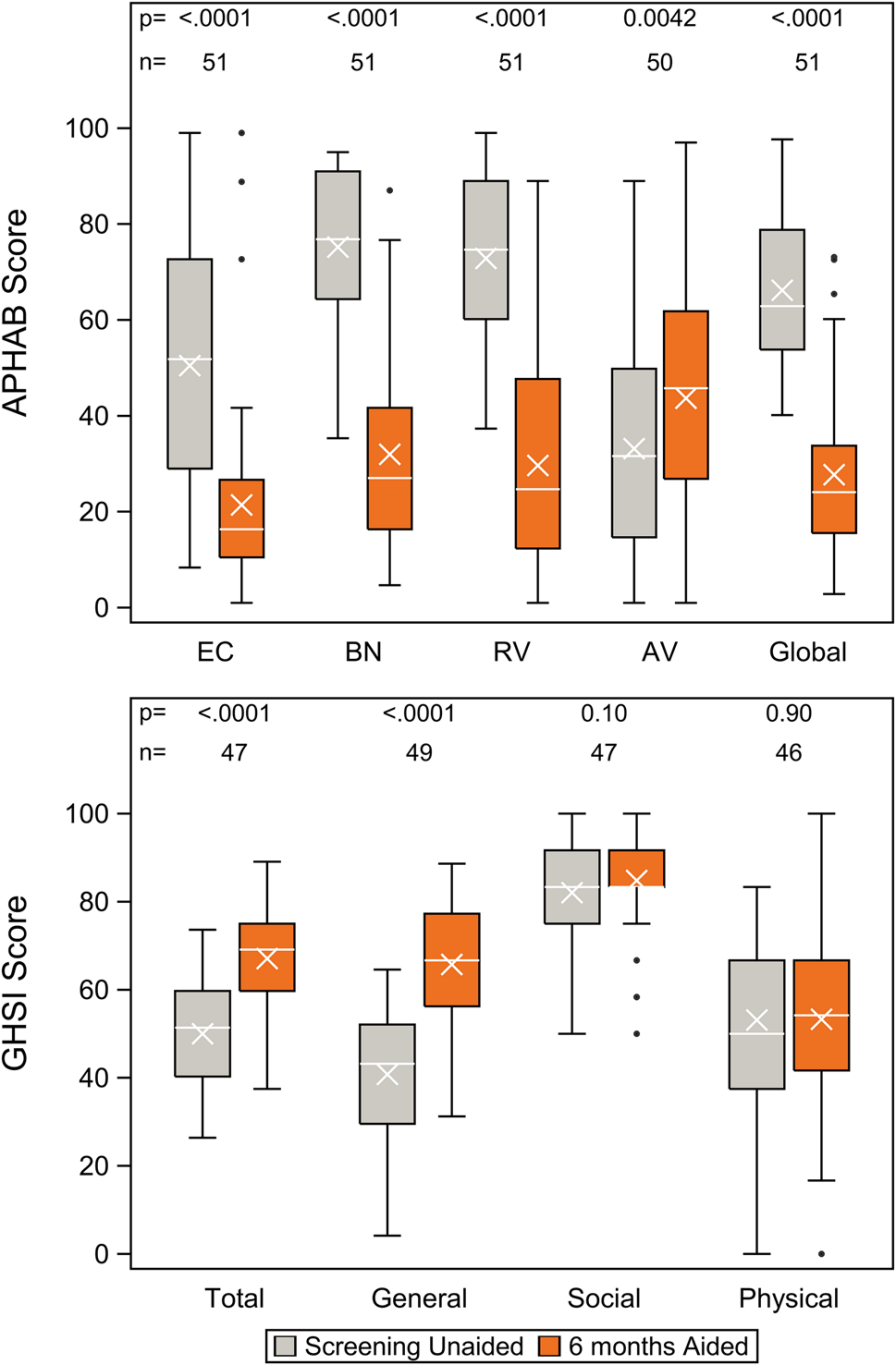
Subjective benefit as measured by the APHAB and GHSI questionnaires, completed before surgery and after 6 months of follow-up. The subscales of the APHAB are represented by the abbreviations on the *x* axis: *EC* ease of communication, *BN* background noise, *RV* reverberation, and *AV* aversiveness of sounds

All patients completed the APHAB and GHSI questionnaires 6 months after surgery. However, 5 patients did not complete baseline questionnaires and were consequently excluded from the benefit analysis. One additional patient did not complete the baseline APHAB, while another 3 patients were excluded from the benefit analysis using the GHSI because of incomplete data on the 6-month questionnaire. The outcomes of these questionnaires are displayed in Fig. 4. For the GHSI, significant improvement was observed for the total and general scores, but not for the social and physical subscales. The APHAB indicated that there was statistically significant improvement on all of the subscales in the aided condition compared to the unaided condition.

## Discussion

The current randomized controlled clinical trial compared outcomes at 6 months of 2 percutaneous bone-anchored hearing implants for bone conduction devices: a new 4.5-mm-wide implant (test) and the 3.75-mm-wide previous generation implant (control), both loaded with the bone conduction device at 3 weeks. The test implant exhibited significantly higher ISQ values than the control implant. All other clinical outcomes were comparable between the implants. Quality of life generally improved in the aided condition compared to before implantation.

The strengths of the current study include the absence of cases lost to follow-up and the conscientiously followed prospective study protocol. The tightly spaced follow-up visits allow for a detailed analysis of the development of the implants’ stability. Therefore, the study design yielded useful information on short-term clinical results for both implants. The study’s strength lies also in the fact that only a single parameter, the implant width/design, was varied. A limitation of the current study was the non-blinded follow-up for the investigators and patients.

Both implants exhibited positive trends in ISQ measurements that generally increased from baseline until the final follow-up at 6 months. These positive trends are an indication of a progression in implant stability over time. RFA application in bone-anchored hearing implants has gained increasing interest in recent years. However, to date, reporting standards vary widely. Therefore, comparisons between different studies should be made very carefully. Foghsgaard and Caye-Thomasen [8] also studied the test implant and found an increasing trend in ISQ in the first year after surgery; however, they noted a slight decrease at the second follow-up visit (mean 7.3 weeks), when loading was applied. In our results, the ISQ was never lower than at surgery. It is worth noting not only that the test implant gave higher ISQ values on average, as expected, but also that the increase in ISQ over time was significantly higher for the test implant than the control implant. Although the present investigation was limited to adult patients with normal bone quality, it might be anticipated that the positive outcomes of the test implant could improve treatment outcomes in pediatric patients and patients with compromised bone quality. In comparable prospective studies on another wide implant type, increasing ISQ trends were reported in the first 6 months as well [10, 11], with a dip in the ISQ at the first follow-up visit after surgery (10 days). A 3-year follow-up on those implants revealed somewhat decreasing trends in ISQs beginning 2 years after implantation [9]. It will be interesting to extend the follow-up period of the current study to observe ISQ trends in comparison.

At this moment, the clinical implications of absolute ISQ values are not yet understood, so only trends should be evaluated. Additionally, in dental implantology, there is still a lack of studies documenting clear clinical benefits from therapeutic decisions based on RFA [13]. The large number of different implant designs in dental implantology might also influence this.

The implant survival rate was 100 % for both implants. The same percentage was reported for the current test implant in another 1-year follow-up prospective case series [8]. An implant survival rate of 96.8 % was reported on the current control implant in a retrospective case series with a mean follow-up period of 16.9 months (range 12.1–25.2 months) [17]. These survival rates are slightly higher than those reported in 2 other prospective studies on a different wide implant type [10, 11]. Although all of these are short-term results, the first year after surgery has been reported to be critical, as more than half of implant losses occur in the first year after surgery [4]. The current study will be extended to compare the results to long-term survival figures from retrospective analyses. Varying survival rates of 81.5–98.4 % with maximum follow-up periods of up to 32.5 years have been reported on previous generation implants (3.75-mm diameter flange fixtures with a design comparable to that of the current control implant) [4–6].

Soft tissue tolerability was comparably good in both the test and control implants, with incidental adverse Holgers grade 2 and 3 skin reactions. This was expected because the abutment, which is believed to mainly influence the skin outcomes, was the same for both the test and control groups. The current adverse soft tissue events are comparable or even slightly better than rates reported from this center in the studies of another type of wide implant [10, 11], also installed with skin thinning techniques. A remarkable fact is that 2 out of 5 patients who presented with adverse skin reactions suffered from skin diseases, which is a higher incidence than in the study population as a whole. This is in agreement with earlier observations [18, 19] and the more recent identification of skin diseases as risk factors for skin reactions around bone-anchored hearing implants in a large retrospective cohort study [den Besten et al. (2014), manuscript accepted for publication in *Otology & Neurotology*].

As both implants were loaded at 3 weeks after implantation, the current study established that early loading did not affect the positive ISQ trend and short-term clinical outcomes. This is confirmed by another study of the current control implant that reported on a loading time as early as 2 weeks after implantation [20]. Early loading of 2, 3, and 4 weeks has also been studied on another type of wide implant with promising short-term results [10, 21, 22].

Hearing-related quality of life improved due to the system as a whole, as patients reported improvements on both the APHAB and GHSI questionnaires from pre-implantation to 6 months later. The aided APHAB outcome is comparable to a similar-sized population with single-sided deafness fitted with bone conduction devices [23] and better than a larger population of elderly patients fitted with bone conduction devices for mixed indications [24]. The APHAB outcome can be strongly influenced by the sound processor used, with modern sound processors producing significantly better aided APHAB scores than older technologies [25]. To our knowledge, the GHSI has not been used to evaluate quality of life improvements with percutaneous bone conduction devices. GBI scores were also positive and comparable between groups. The current GBI outcome compares positively to other studies that used the GBI to establish benefit from bone conduction systems (see Table 3 in Faber et al. [10]). It should be emphasized that indications and patient characteristics influence quality of life, so comparisons with these other studies should be made carefully. Intra-study comparisons of aided vs unaided conditions are, therefore, more important than inter-study comparisons.

## Conclusion

After 6 months of follow-up, outcomes of a new 4.5-mm diameter percutaneous implant for bone conduction devices compared to the previous generation 3.75-mm diameter implant exhibited higher ISQ values and similarly promising clinical characteristics. No implants were lost, and soft tissue tolerability was good. Loading both implants at 3 weeks appeared to be safe and hearing-related quality of life improved. These positive short-term results indicate that the new implant and its corresponding hearing devices loaded at 3 weeks is a safe option for hearing rehabilitation.
